# Comprehensive Identification and Integrated Analysis of the *PR1* and *PR10* Gene Families in Ginseng: *PgPR1-5* and *PgPR10-8* Gene Expression Analysis in Response to MeJA

**DOI:** 10.3390/life16091446

**Published:** 2026-08-30

**Authors:** Kexin Zhang, Jiaxiang Feng, Meiyan Fan, Yu Zhang, Mingzhu Zhao, Meiping Zhang, Yi Wang, Lei Zhu, Kangyu Wang

**Affiliations:** 1 College of Life Science, Jilin Agricultural University, Changchun 130118, China; zkx12302023@163.com (K.Z.); fjx1531@163.com (J.F.); fmy18247587123@163.com (M.F.); yuzi_zhang0913@163.com (Y.Z.); mingzhuzhao@jlau.edu.cn (M.Z.); meiping.zhang@jlau.edu.cn (M.Z.); yi.wang@jlau.edu.cn (Y.W.); 2 Jilin Engineering Research Center Ginseng Genetic Resources Development and Utilization, Jilin Agricultural University, Changchun 130118, China

**Keywords:** *Panax ginseng*, *PgPR1* gene family, *PgPR10* gene family, methyl jasmonate, genes expression pattern

## Abstract

Pathogenesis-related (PR) proteins are central to plant defense; however, their roles in regulating secondary metabolism remain largely unexplored. In *Panax ginseng*, the molecular characteristics and functional links of *PR1* and *PR10* gene families to ginsenoside biosynthesis remain unclear. Here, we conducted genome and transcriptome identification, evolutionary analysis, co-expression network construction, and methyl jasmonate (MeJA) induction assays to investigate their roles. We identified 34 *PR1/PR10* family members (11 *PgPR1s* and 23 *PgPR10s*) unevenly distributed across 15 chromosomes, with gene duplication driving family expansion. Functional divergence was evident: PgPR1 members are evolutionarily conserved and root-preferential, associated with antibacterial defense, whereas PgPR10 underwent species-specific expansion, showed broad tissue expression, and possessed promoters enriched with hormone- and stress-responsive *cis*-elements. Co-expression analysis (|*r*| ≥ 0.3, *p* < 0.001) identified *PgPR1-5* and *PgPR10-8* as the only *PR* members significantly correlated with key ginsenoside biosynthetic enzymes. Under MeJA, the ginsenoside pathway displayed bidirectional temporal regulation, with core *PR* genes showing synchronized expression with the negatively regulated enzyme gene *CAS_14*. Our study characterizes the molecular and evolutionary features of PgPR1/PgPR10 families, identifies core *PR* genes associated with ginsenoside metabolism, and reveals their coordinated response to MeJA, providing key insights and candidate targets for exploring the potential link between PR proteins and ginsenoside biosynthesis and promising candidate genes for further research on *P. ginseng*.

## 1. Introduction

*Panax ginseng*, a perennial herb of the genus *Panax* in the family Araliaceae, is naturally distributed in temperate forests where it grows under the forest canopy, and has long adapted to the low-light, low-temperature, and high-humidity shaded environment [1,2]. It represents one of the medicinal plants with a relatively well-resolved genome among non-model plant species [3,4]. By continuously accumulating high concentrations of ginsenosides (which can account for 2–7% of the dry weight in the root, and exceed 5% under certain conditions or in wild populations), *P. ginseng* establishes a constitutive chemical defense barrier [5,6,7,8,9]. This trade-off between slow growth and high chemical defense represents a crucial adaptation mechanism of perennial shade-dwelling plants to resource-limited environments [10]. However, the molecular regulatory basis underlying this adaptation, particularly whether the PR protein families are functionally associated with the ginsenoside biosynthetic pathway, remains poorly understood and requires detailed investigation.

Pathogenesis-related (PR) proteins are a class of defense proteins specifically produced in higher plants following pathogen infection or elicitor treatment [11]. They are widely present in higher plants and play a central role in systemic acquired resistance (SAR) and induced systemic resistance (ISR) [12]. Based on their structural characteristics, serological relationships, and biological functions, PR proteins are currently classified into 17 families (PR1–PR17). Among these, PR1 and PR10 are the two most extensively studied families and are known to participate in biotic stress responses across a wide range of plant species [13,14]. PR1 proteins exhibit antifungal activity and are recognized as biomarkers of SAR, with their accumulation levels positively correlated with plant resistance to pathogens. Members of this family typically contain a conserved cysteine-rich antimicrobial protein (CAP) domain, which is rich in cysteine residues and forms a stable three-dimensional conformation capable of binding to fungal membrane sterols, thereby exerting antimicrobial functions [15]. PR10 proteins possess both ribonuclease activity and stress response functions, participating in both biotic and abiotic stress responses across diverse plant species. Their characteristic structure is the Bet-v-1 (Betula verrucosa allergen 1) domain, which consists of seven antiparallel β-sheets and two α-helices, forming a hydrophobic ligand-binding cavity. This cavity can bind molecules such as cytokinins, fatty acids, and flavonoids, suggesting that PR10 proteins may be involved in signal transduction or metabolic regulation through ligand binding [16,17,18].

It is noteworthy that PR protein expression is often stringently regulated by the jasmonic acid (JA) signaling pathway [19,20]. Methyl Jasmonate (MeJA), a volatile derivative of jasmonic acid, can effectively mimic biotic stress signals when applied exogenously, thereby efficiently inducing the activation of plant defense responses [21]. In *P. ginseng*, previous studies have demonstrated that MeJA treatment not only significantly promotes the accumulation of ginsenosides but also induces the expression of multiple defense-related genes [22,23,24]. This suggests that MeJA may act as a key signaling hub coordinating the synergistic interplay between defense responses and secondary metabolism in ginseng. However, comprehensive genome and transcriptome identification, evolutionary characterization, and analysis of the expression regulation patterns of the *PR1* and *PR10* gene families in *P. ginseng* under MeJA signaling induction remain incomplete. In particular, systematic research utilizing co-expression network analysis to screen *PR* candidate genes functionally linked to key ginsenoside biosynthetic enzyme genes has yet to be conducted.

Therefore, based on the genomic data of *P. ginseng*, this study conducted a genome and transcriptome identification of the *PgPR1* and *PgPR10* gene families and systematically analyzed their evolutionary characteristics, conserved domains, and cis-acting elements in promoter regions. Combined with transcriptomic data, the differential expression patterns of these genes under MeJA induction were investigated. Furthermore, through gene co-expression network analysis, candidate *PR1* and *PR10* genes highly associated with key ginsenoside biosynthetic enzyme genes were screened, and the potential molecular mechanisms by which PR proteins participate in the synergistic regulation of defense responses and secondary metabolism in ginseng were preliminarily elucidated. The findings of this study provide a theoretical basis for understanding the molecular mechanisms underlying the defense–growth trade-off in perennial shade-dwelling medicinal plants and offer valuable candidate gene resources for disease-resistant breeding and quality improvement in *P. ginseng*.

## 2. Materials and Methods

### 2.1. Comprehensive Identification of PR1 and PR10 Gene Families in P. ginseng

This study used de novo assembled transcriptomic unigene resources of *P. ginseng* without reference genomic data, and performed a transcriptome-wide scan of translated unigene protein sequences using the HMMER version 3.0 algorithm to screen candidate sequences carrying PR1 (PF00188) or PR10 (PF00407) structural domains (E-value < 1 × 10^−6^). Subsequently, the integrity of conserved domains was verified using the SMART online tool (https://smart.embl.de/, accessed on 5 January 2026); NCBI ORF Finder was additionally applied to filter transcripts with premature stop codons and incomplete open reading frames. All transcripts with intact characteristic domains were retained without removing homologous or alternative splicing isoforms, and all qualified sequences were systematically named as final *PgPR1* and *PgPR10* family genes.

### 2.2. Phylogenetic, Conserved Domain and Motif Analysis of PR1 and PR10 Gene Families in Ginseng

To achieve systematic classification and characterization of the *PR1* and *PR10* gene families in *P. ginseng*, this study first screened *PgPR1* and *PgPR10* members carrying complete open reading frames (ORFs) as core research objects; to carry out multi-angle evolutionary comparison, we selected representative species covering different plant evolutionary lineages for comparative genomic analysis with distinct screening purposes for the two families. Specifically, for the *PR1* family, rice (*Oryza sativa*, monocot model), *Arabidopsis thaliana* and tomato (*Solanum lycopersicum*, typical dicot models) were selected to compare *PR1* evolutionary divergence between monocots and dicots, and their homologous sequences were aligned with *PgPR1* genes. For the *PR10* family, multiple representative species were included for comprehensive analysis: the monocot model durum wheat (*Triticum durum*) and dicot model *Arabidopsis thaliana* for cross-lineage comparison; carrot (*Daucus carota*), tobacco (*Nicotiana tabacum*), soybean (*Glycine max*) and barrel medic (*Medicago truncatula*) to explore *PR10* functional variation in plants with diverse secondary metabolic characteristics; and the closely related congeneric species *Panax notoginseng* to reveal lineage-specific expansion patterns of the *PR10* family within the Araliaceae, with all these homologous sequences analyzed together with *PgPR10* genes. Phylogenetic trees were constructed using MEGA version X software, with clustering performed using the neighbor-joining method. The robustness and reliability of the tree topology were assessed by setting bootstrap replicates to 1000 for validation through repeated sampling [25]. Subsequently, the online visualization platform iTOL (https://itol.embl.de/itol_account.cgi, accessed on 1 August 2026) was employed to perform refined beautification and structural optimization of the constructed phylogenetic trees, clearly displaying the clustering characteristics of family members [26]. Furthermore, online analysis tools were employed to identify and characterize the conserved domains and motifs of the PgPR1 and PgPR10 protein families in ginseng. A total of five conserved motifs (Motif 1 to Motif 5) were successfully identified and visualized. Additionally, the distribution patterns of the *PgPR1* family-specific CAP superfamily and CAP_PR-1 domains, as well as the *PgPR10* family-specific Bet_v1-like domain and Bet_v_1 domain, were determined across all family members. This analysis provides fundamental data for elucidating the structural characteristics and functional differentiation of the two gene families.

### 2.3. Chromosomal Localization and Collinearity Analysis of PR1 and PR10 Gene Families

Based on the identified sequences of the *PR1* and *PR10* gene families in *P. ginseng*, the BLASTN algorithm was used to align them with the ginseng reference genome. The screening thresholds were set as follows: sequence identity ≥ 95%, alignment coverage ≥ 200 bp, and E-value ≤ 1.0 × 10^−100^. After extracting the chromosome physical location information, the MG2C v2.1 online platform (http://mg2c.iask.in/mg2c_v2.1/, accessed on 21 January 2026) was used to generate a visual distribution map of the genes across the 24 chromosomes [27]. For the members of the *PR1* and *PR10* gene families, based on the chromosomal localization data described above, intraspecific collinearity Circos plots were constructed using the R package circlize (v4.2.0). By analyzing the collinear relationships of gene pairs between chromosomes, the characteristics of gene duplication events in the *PR1* and *PR10* gene families and their distribution patterns at the chromosomal level were elucidated.

### 2.4. Promoter Cis-Acting Elements Analysis of the PR1 and PR10 Gene Family in P. ginseng

To investigate the potential biological functions of the *PR1* and *PR10* gene families, promoter sequences spanning 2000 bp upstream of the start codon of each gene were extracted based on their chromosomal localization information. These promoter sequences were subjected to comprehensive scanning and identification of cis-acting regulatory elements using the PlantCARE online analysis platform [28]. After identification, cis-acting elements associated with hormone responsiveness were screened, and their distribution patterns within the promoter regions were visualized and analyzed using R (v4.0) software. This study aimed to elucidate the potential regulatory mechanisms of the *PR1* and *PR10* gene families in plant hormone signaling pathways.

### 2.5. Expression Profiling of PR1 and PR10 Gene Families in P. ginseng

Expression data for the *PR1* and *PR10* gene families were extracted from three types of samples, including the 4 different ages (5, 12, 18, 25 years old) of ginseng roots, the 14 different tissues of 4-year-old ginseng, and 42 farmers’ cultivars of 4-year-old ginseng roots, based on the transcriptomic expression database of *P. ginseng*. After removing transcripts with no detectable expression, data normalization and visualization were performed using R software, and expression heatmaps were constructed using TBtools II version 2.402 to illustrate the tissue-specific expression profiles and inter-varietal expression variations in the *PR1* and *PR10* gene families [29].

### 2.6. Gene Co-Expression Network Construction for PR1 and PR10 Families in P. ginseng

To further investigate the interaction characteristics of *PR1* and *PR10* gene expression among 42 farmer cultivars of *P. ginseng*, Spearman’s correlation coefficient was calculated using the SPSS version 23.0 software to evaluate the correlations of gene expression levels. Based on the correlation coefficient matrix, gene pairs with significant correlations were screened with thresholds of |*r*| ≥ 0.3 and *p* < 0.05. Co-expression networks were constructed separately for the *PR1*, *PR10*, and merged *PR1* and *PR10* gene family datasets using the CNSknowall online analysis platform (https://cnsknowall.com, accessed on 1 August 2026). In the networks, the node size was weighted according to the degree value, the thickness of the edges reflected the absolute value of the correlation coefficient, and red and blue represented positive and negative correlations, respectively. This analysis helps reveal the intermember interaction relationships within the *PR1* and *PR10* gene families, the cross-family expression associations, and their potential functional connections between the two gene families. A relatively lenient significance threshold of *p* < 0.05 was adopted here to retain all moderate expression correlations within the *PR1*/*PR10* families and fully display the internal coordination patterns among homologous *PR* genes.

### 2.7. Identification of Ginsenoside Biosynthesis-Related Candidate Genes from PR1 and PR10 Gene Families in P. ginseng

Based on the transcriptomic expression data of 42 farmer cultivars of ginseng, this study systematically compiled the expression profiles of *PR1* and *PR10* genes along with 20 key ginsenoside biosynthetic enzyme genes. Pearson’s correlation analysis was performed using the SPSS version 23.0 software, with a significance threshold set at *r* ≥ 0.3 and *p* < 0.001. Co-expression networks were constructed for the *PR1* gene family, the *PR10* gene family, and the combined dataset of both families using the CNSknowall online analysis platform. In the resulting networks, node sizes were weighted according to the degree of connectivity, edge thickness represented the absolute value of the correlation coefficient, and red and blue colors indicated positive and negative correlations, respectively. This approach visually reveals the expression association patterns between members of the *PR1* and *PR10* gene families and key ginsenoside biosynthetic enzyme genes. To strictly reduce false-positive associations and reliably screen core *PR* genes tightly linked to ginsenoside biosynthesis, a more stringent threshold of *p* < 0.001 was used for the correlation analysis between *PR* genes and ginsenoside biosynthetic enzyme genes, while maintaining the same correlation coefficient cutoff |*r*| ≥ 0.3 as Section 2.6 for consistency.

### 2.8. MeJA-Elicited Expression Changes in Ginsenoside Biosynthetic Enzyme Genes and PR1/PR10 Gene Families in Ginseng Adventitious Roots

The ginseng adventitious roots used in this study were induced from ginseng seedlings and provided by the Jilin Engineering Research Center for Ginseng Genetic Resources Development and Utilization. The adventitious roots were subcultured on B5 medium supplemented with 500 mg/L IBA. Medium pH was adjusted to 6.0, and cultures were maintained under continuous darkness. Approximately 1.0 g of adventitious root material was inoculated into a 250 mL conical flask containing 150 mL liquid MS medium and incubated on a rotary shaker at 110 rpm and 22 °C for 21 days of preculture. Each independently cultured conical flask was defined as one biological replicate.

Subsequently, exogenous treatment was performed with 200 μM MeJA, a concentration optimized in our laboratory. MeJA stock solution was prepared with absolute ethanol, and the final concentration of ethanol in the medium was lower than 0.1% (*v*/*v*). Sampling was conducted across 10 time points: 0 (untreated initial control), 6, 12, 24, 36, 48, 60, 72, 84, and 96 h. Three biological replicates (independent flasks) were prepared for each time point. The total culture duration spanned 25 days, and adventitious root samples were harvested at each designated time point. Total RNA was extracted from 0.1 g adventitious root tissue using TRIzol reagent (CWBIO, Beijing, China). RNA was reverse-transcribed into cDNA with the RevertAid™ First Strand cDNA Synthesis Kit (MBI). *Actin 1* was selected as the reference gene. Preliminary stability tests in our lab confirmed that *Actin 1* exhibits consistently stable transcript abundance across all MeJA treatment time points of ginseng adventitious roots. Consistent with multiple published RT-qPCR studies focused on MeJA-mediated ginsenoside biosynthesis, this single stable reference gene was applied for expression normalization. Moreover, three biological replicates and three technical replicates were set for all samples to minimize analytical bias caused by using only one reference gene. Real-time quantitative PCR (RT-qPCR) was carried out on a 7500 Real-Time PCR System using the UltraSYBR One-step RT-qPCR Kit (Low ROX) (CWBIO, Beijing, China) to detect expression patterns of key ginsenoside biosynthetic enzyme genes (*CYP716A53v2_1*, *CYP716A52v2_3*, *CYP716A47_1*, *SS_1*, *SE2_1*, *SE2_4*, *DS_1*, *DS_3*, *FPS_22*, *UGT71A27_2*, and *CAS_14*) and pathogenesis-related protein genes (*PR1* and *PR10*). In this study, the *Actin 1* gene was used as the reference gene, and all the primers used and their sequences are listed in Table 1. The total volume of the RT-qPCR reaction system was 10 μL, consisting of 5 μL UltraSYBR Mix (Low ROX), 0.2 μL each of forward and reverse primers (10 μM), 1 μL cDNA template, and 3.6 μL ddH_2_O. The amplification program was as follows: 95 °C for 10 min; followed by 40 cycles of 95 °C for 15 s, 60 °C for 60 s, and 72 °C for 30 s. Each sample was analyzed with three biological replicates and three technical replicates, and relative gene expression levels were calculated using the 2^−ΔΔCt^ method.

## 3. Results

### 3.1. Identification of PR (Pathogenesis-Related) Protein Gene Families in P. ginseng

In the ginseng genome and transcriptome databases, 34 genes belonging to the *PR1* and *PR10* families were identified, including 11 *PgPR1* gene family members and 23 *PgPR10* gene family members. Based on conserved domain features, these genes can be divided into two categories: all PR1 family members contain CAP domains, whereas PR10 family members contain Bet_v_1 and Bet_v_1-like domains. They were sequentially named PgPR1-1 to PgPR1-11 and PgPR10-1 to PgPR10-23 (Table S1). Supplementary Table S1 provides detailed information for all genes, including gene name, gene ID, transcript length (bp), nucleic acid sequence (5′–3′), open reading frame start and stop (ORF, bp), and predicted amino acid sequence (5′–3′).

### 3.2. Phylogenetic and Structural Analysis of PR1 and PR10 Gene Family

**Figure 1:**
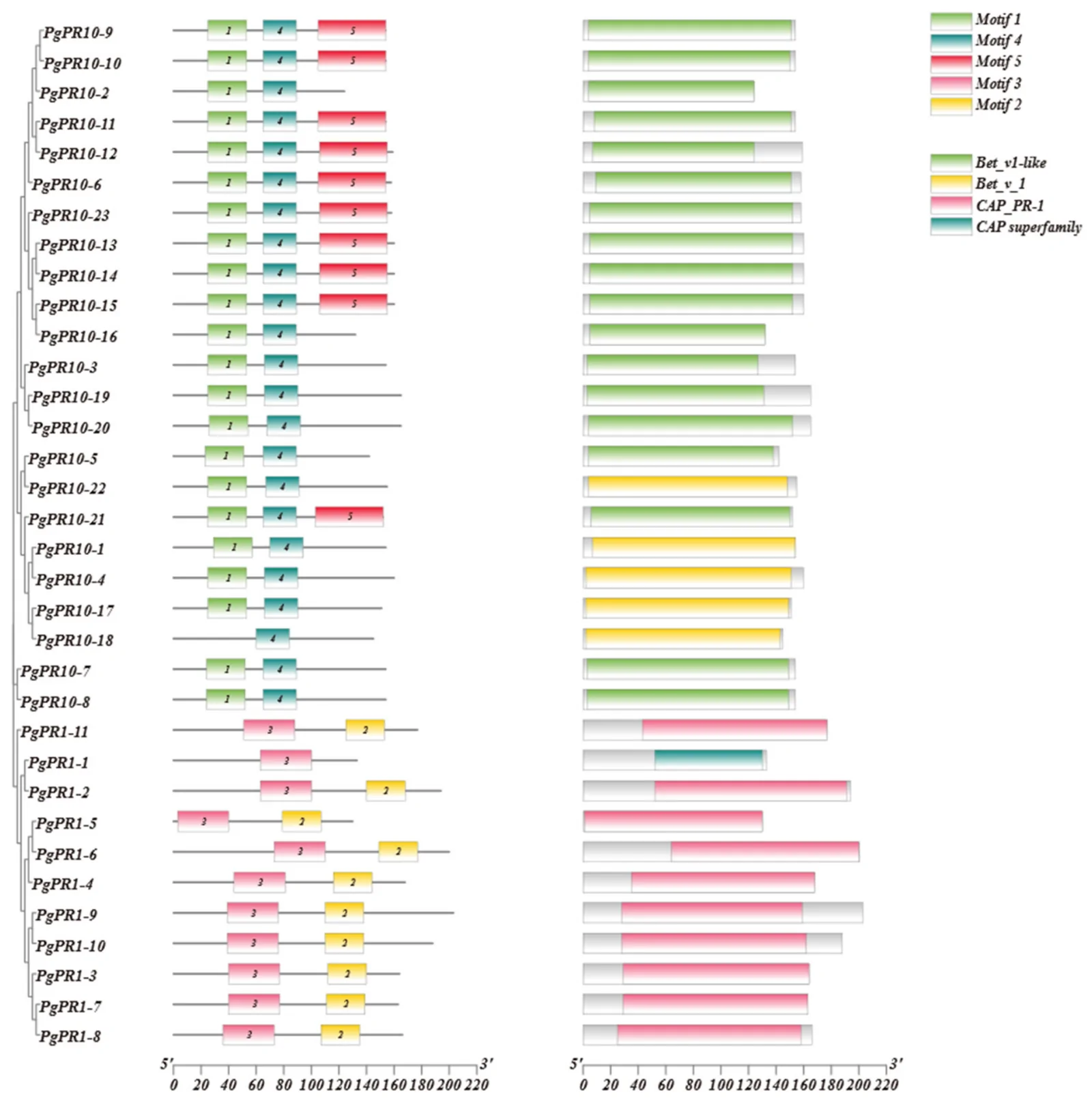
Distribution of conserved motifs and characteristic domains in PgPR1 and PgPR10 protein gene families in *P. ginseng*. The abscissa represents the amino acid sequence length (aa) of proteins, and the ordinate represents each member of the gene families. The PgPR1 family is characterized by CAP superfamily and CAP_PR-1 domains, while the PgPR10 family contains Bet_v1-like and Bet_v_1 domains. Different conserved motifs are arranged in a species-specific and orderly manner in the two families without cross-distribution, reflecting the significant structural differentiation of their proteins.

As shown in Figure 1, the distribution of conserved motifs and domains of PR1/PR10 family proteins in *P. ginseng* exhibits obvious family specificity, and the types of domains within the same family are highly uniform. All PR1 family members contained the CAP superfamily and CAP_PR-1 conserved domains, whereas PR10 family members possessed either Bet_v_1 or Bet_v_1-like conserved domains. No overlapping domain distribution was observed between the two families, reflecting significant structural differentiation between the PR1 and PR10 families. In total, five conserved motifs (Motif 1–Motif 5) were identified in PR1/PR10 family proteins in *P. ginseng*. The distribution of these five conserved motifs exhibited a certain regularity within each family, and the motif composition differed significantly between the PR1 and PR10 families, further reflecting the specificity in functional architecture between the two families. In terms of physical location, these motifs were arranged in an orderly manner from the N-terminus to the C-terminus of the protein sequences without an obvious random distribution, and the protein lengths were mostly within 220 aa, indicating a relatively compact structural feature.

**Figure 2:**
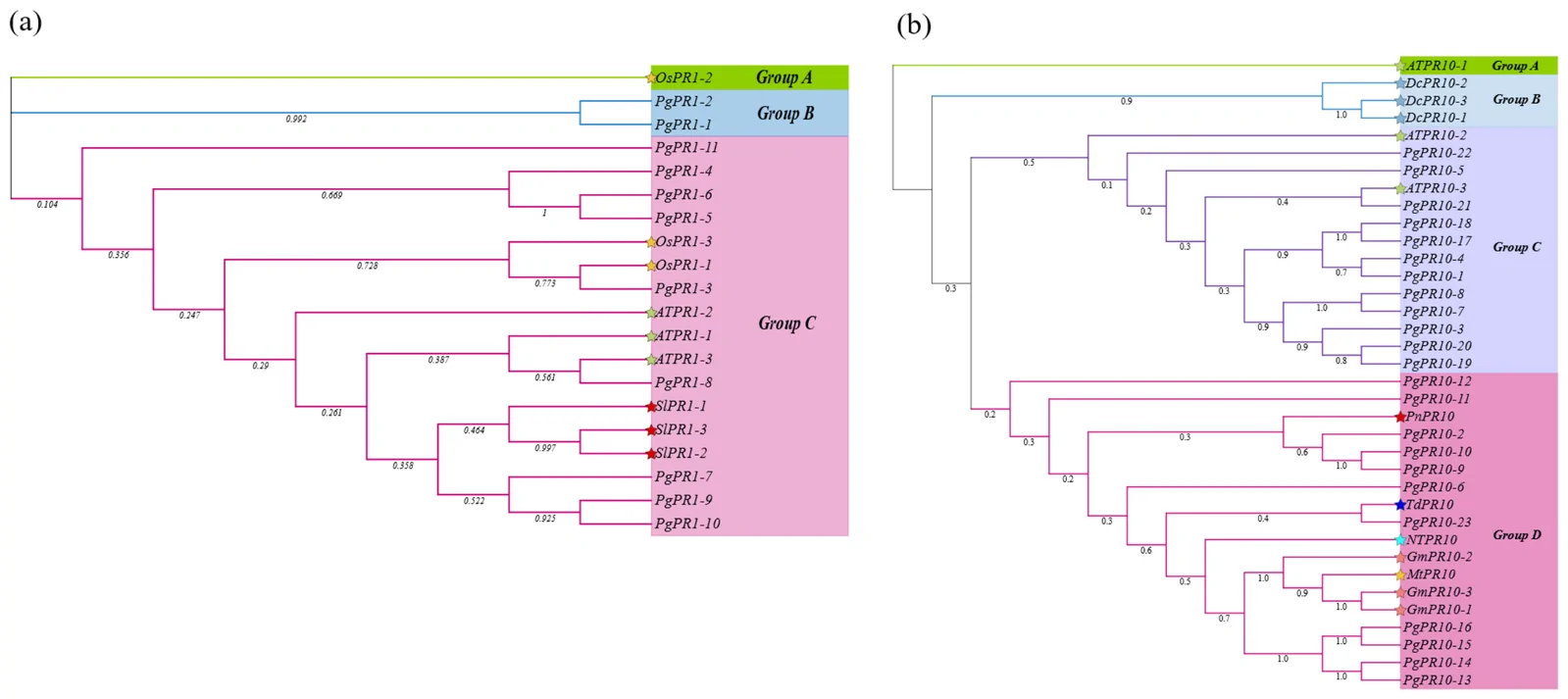
The *PR1* and *PR10* gene families phylogenetic tree analysis. (**a**) Phylogenetic tree of *PR1* family members from *P. ginseng* (*PgPR1*), *O. sativa* (*OsPR1*), *A. thaliana* (*AtPR1*) and *S. lycopersicum* (*SlPR1*) constructed by Neighbor-Joining (NJ) method; (**b**) Phylogenetic tree of *PR10* family members from *P. ginseng* (*PgPR10*), *A. thaliana* (*AtPR10*), *D. carota* (*DcPR10*), *T. durum* (*TdPR10*), *N. tabacum* (*NtPR10*), *G. max* (*GmPR10*), *M. truncatula* (*MtPR10*) and *P. notoginseng* (*PnPR10*). The bootstrap test was repeated 1000, and the values on the branches represent confidence levels. The *PR1* family was clustered into three branches, showing cross-species distribution and monocot-dicot evolution characteristics; the *PR10* family was clustered into four branches, and PgPR10 members showed species-specific expansion and cross-species clustering in Groups C and D.

Phylogenetic analysis clustered ginseng PgPR1 proteins with their homologous counterparts from rice (OsPR1), *Arabidopsis* (AtPR1), and tomato (SlPR1) into three distinct clades (Group A–C; Figure 2a). The cross-species distribution of homologous sequences within each clade suggests that the core structural characteristics of the PR1 family were already established before the evolutionary separation of monocot and dicot plants. Group A formed a monocot-specific lineage consisting only of rice OsPR1-2, while Group B contained two ginseng-specific isoforms, PgPR1-1 and PgPR1-2, which clustered into a well-supported species-specific subclade (bootstrap = 0.992) and formed a distinct monophyletic lineage in ginseng. Notably, the deep node linking Group B to Group A and Group C showed very low bootstrap support (bootstrap = 0.104), prohibiting reliable inference regarding the early divergence of these lineages. As the largest clade, Group C accommodated the remaining PR1 members from all four species, with its internal sequences clearly divided into monocot and dicot subgroups, reflecting long-term evolutionary divergence between the two plant groups; however, low statistical support at the basal nodes restricts further exploration of the early evolutionary processes underlying this lineage.

As shown in Figure 2b, the PR10 family in *P. ginseng* (PgPR10) clustered into four major evolutionary clades (Groups A–D) together with orthologs from *Arabidopsis thaliana* (*AtPR10*), *Daucus carota* (*DcPR10*), *Nicotiana tabacum* (*NtPR10*), *Panax notoginseng* (*PnPR10*), *Triticum durum* (*TdPR10*), *Glycine max* (*GmPR10*), and *Medicago truncatula* (*MtPR10*). Group A corresponds to an *Arabidopsis*-specific branch containing only *AtPR10-1*. Group B is a carrot-specific clade consisting of three *DcPR10* members (*DcPR10-1*, *DcPR10-2*, and *DcPR10-3*); *DcPR10-1* and *DcPR10-3* share the closest phylogenetic relationship (bootstrap = 1.0). Group C harbors the largest number of *PgPR10* members, with 12 *PgPR10* genes (50% of the whole gene family) grouping alongside *AtPR10-2* and *AtPR10-3*. Within this clade, *PgPR10-21* closely clusters with *AtPR10-3*, while *PgPR10-22* and *PgPR10-5* form a related branch with *AtPR10-2*, indicating considerable sequence homology between these ginseng proteins and *Arabidopsis PR10* homologs. Group D constitutes the largest clade on the phylogenetic tree and displays mixed cross-species distribution, including homologs from ginseng, tobacco, *P. notoginseng*, durum wheat, soybean and *Medicago*. *PgPR10* members are scattered throughout Group D and alternately cluster with orthologs from other species. For instance, *P. notoginseng PnPR10* forms a tight subclade with *PgPR10-9* and *PgPR10-10*; *PgPR10-23* clusters with durum wheat *TdPR10*; *PgPR10-16*, *PgPR10-15*, *PgPR10-14* and *PgPR10-13* group separately with soybean *GmPR10* and *Medicago MtPR10* genes. The topology reveals that ginseng *PR10* genes within Group D form sister lineages with *PR10* homologs from diverse plant species.

### 3.3. Chromosomal Localization and Synteny Analysis of PR1/PR10 Families in P. ginseng

**Figure 3:**
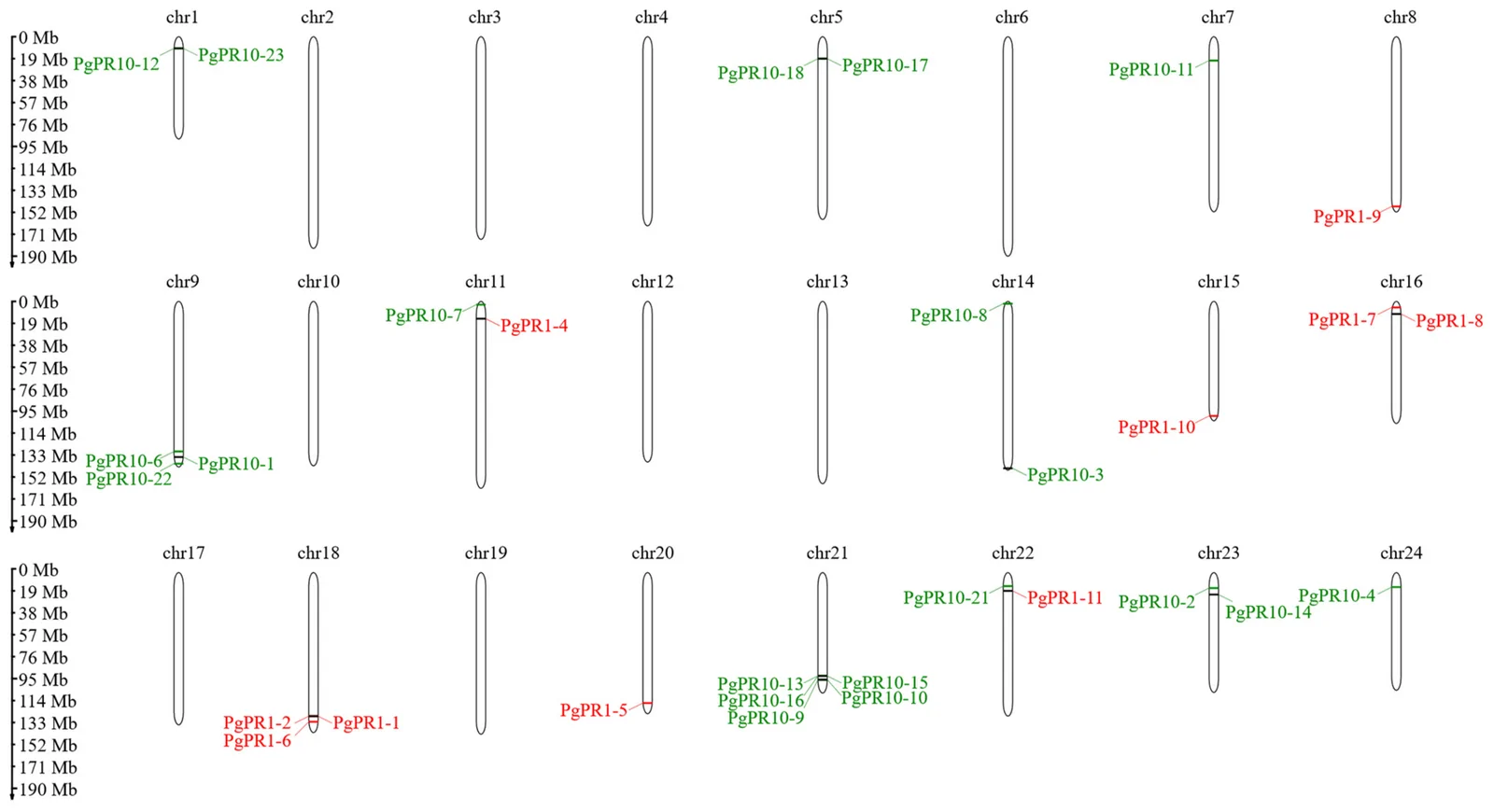
Chromosomal localization and gene cluster distribution of *PgPR1* and *PgPR10* gene families in *P. ginseng*. The physical localization map of *PgPR1* (red) and *PgPR10* (green) family members on 24 chromosomes, with the abscissa representing chromosomal physical length (Mb). Ten annotatable *PgPR1* members were distributed on 7 chromosomes and formed a gene cluster on chr18; twenty annotatable *PgPR10* members were distributed on 10 chromosomes with a terminal enrichment characteristic, forming core gene clusters on chr9 and chr21, and another 3 members were not annotated to specific chromosomes.

Based on chromosomal localization analysis (Figure 3), members of the *PR1*/*PR10* gene families in *P. ginseng* were widely distributed across 15 chromosomes. Among them, 10 of the 11 members in the *PR1* gene family (marked in red), excluding *PgPR1-3*, which was not annotated to any chromosome, and were distributed on seven chromosomes (chr8, 11, 15, 16, 18, 20, 22), and a gene cluster composed of *PgPR1-1*, *PgPR1-2*, and *PgPR1-6* was present on chromosome 18. For the *PR10* gene family (marked in green), 20 of the 23 members, excluding *PgPR10-5*, *PgPR10-19*, and *PgPR10-20*, which were not annotated to any chromosome, and were distributed on 10 chromosomes (chr1, 5, 7, 9, 11, 14, 21, 22, 23, 24), showing an obvious telomeric enrichment pattern, with distinct gene clusters formed on chr21 (containing five members) and chr9 (containing three members).

**Figure 4:**
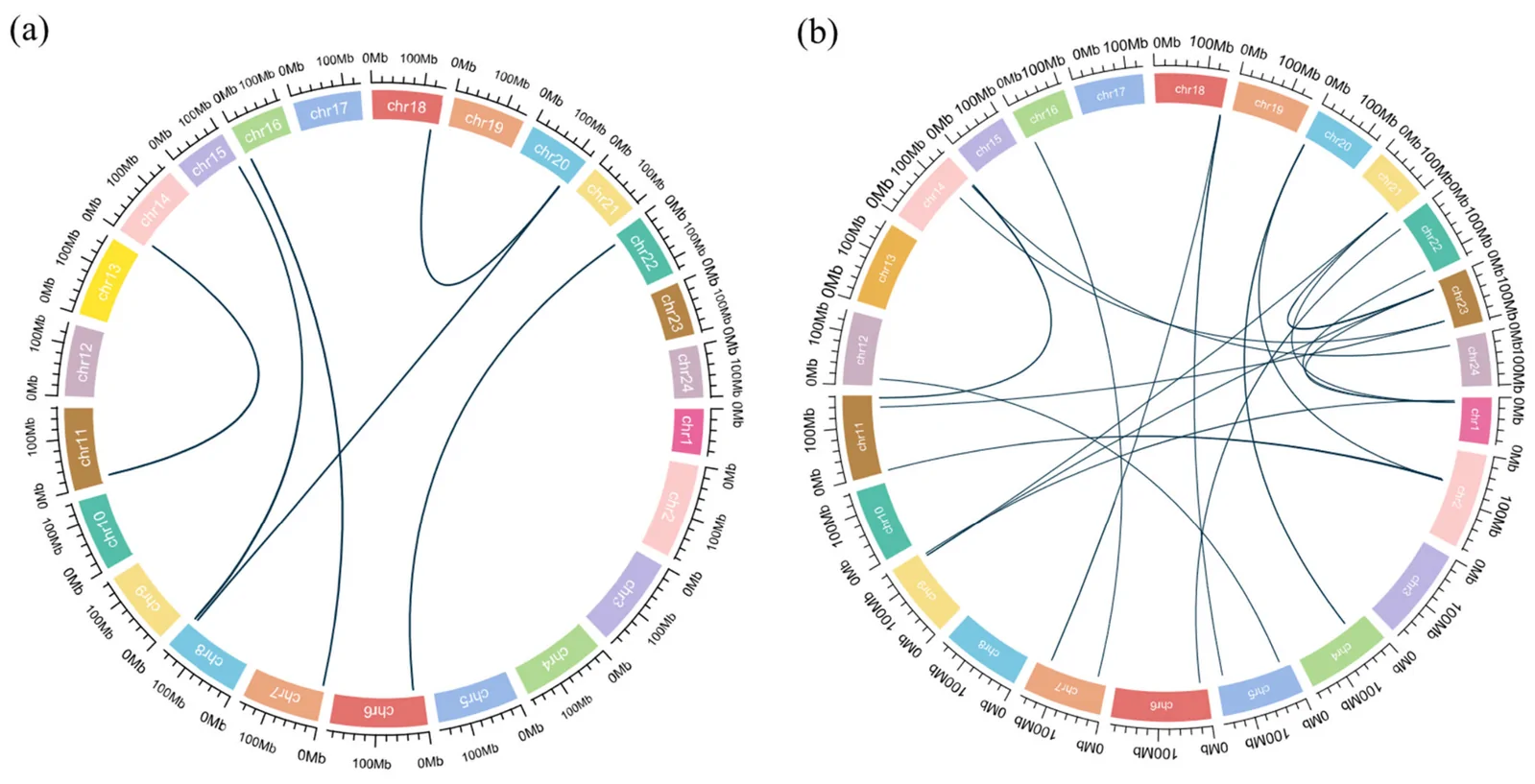
Circular plot showing the chromosomal distribution and homologous pairwise relationships of *PgPR1* and *PgPR10* gene families in the ginseng genome. (**a**) Homologous relationships of the *PgPR1* family; six inter-chromosomal homologous gene pairs are displayed with non-intersecting connecting lines. (**b**) Homologous relationships of the *PgPR10* family; nineteen homologous gene pairs are distributed across 17 chromosomes. Chromosomes 21, 22 and 23 contain abundant homologous connections, and long-distance intrachromosomal homology can be observed. Circular tracks correspond to ginseng chromosomes, and internal lines represent pairwise homologous gene relationships.

Homologous pairwise relationships within the *PR1* family in ginseng revealed six gene pairs whose homologous members are located on different chromosomes (Figure 4a). These inter-chromosomal homologies are presented as discrete, non-intersecting links in the circular plot. For the *PR10* family, a more intricate network of homologous gene pairs was identified, consisting of 19 homologous pairs distributed across 17 chromosomes. Notably, chromosomes 21, 22, and 23 displayed a higher density of such homologous connections. Long-distance homologous relationships were detected between chromosomes, including chr1–chr21 and chr5–chr23. Combined with chromosomal localization data, the five-gene cluster on chr21 and the three-gene cluster on chr9 both connect to multiple homologous partners, reflecting extensive sequence similarity among these genomic loci (Figure 4b).

### 3.4. Cis-Acting Elements Promoter Analysis of PgPR1 and PgPR10 Gene Families

**Figure 5:**
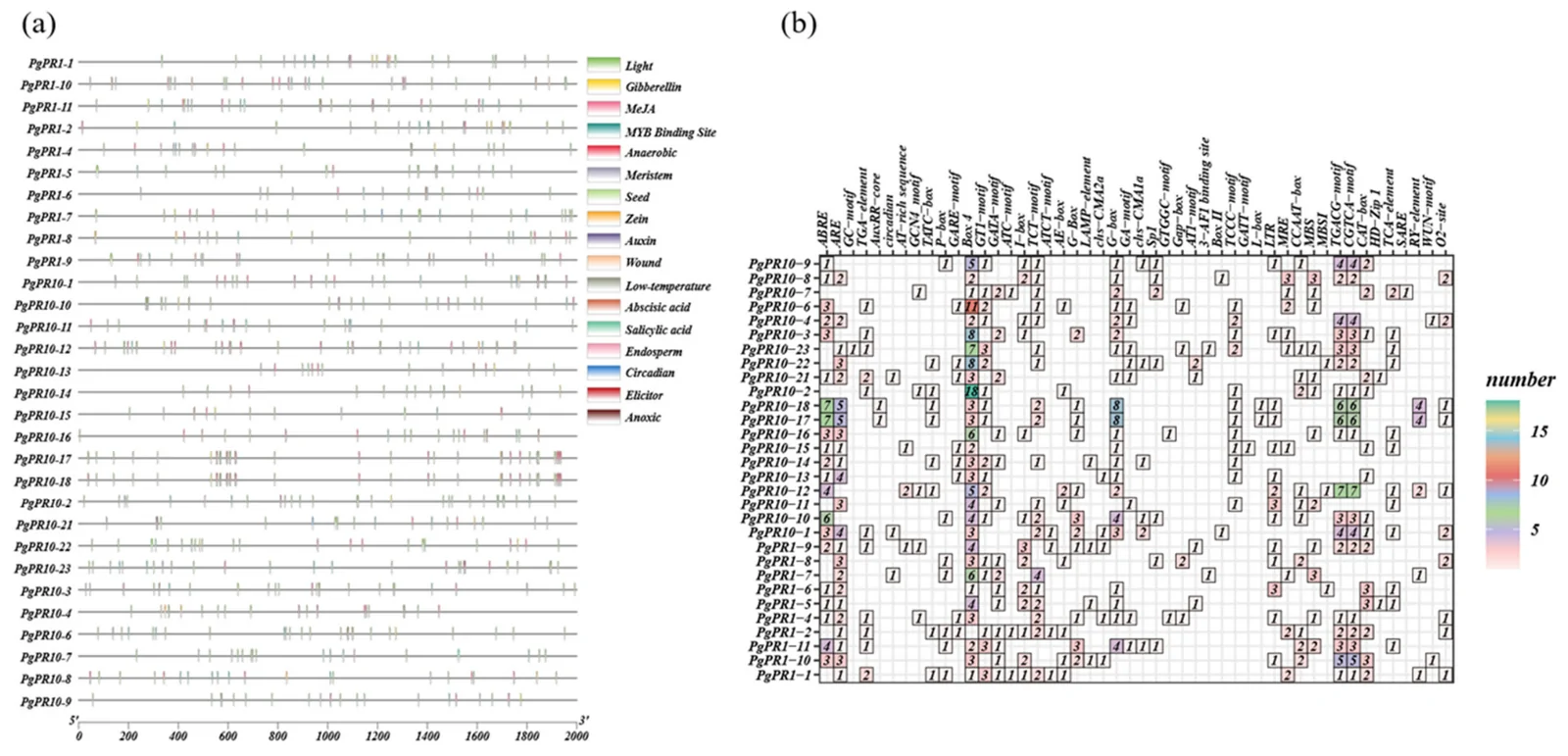
Distribution and quantity characteristics of cis-acting elements in the promoter regions of *PgPR1* and *PgPR10* genes in ginseng. (**a**) Localization map of cis-acting elements in the promoter region upstream of the start codon of each *PgPR1* and *PgPR10* member, with the abscissa representing promoter sequence length (bp). The elements include light response, hormone response, abiotic stress response and tissue development regulation types; (**b**) Heatmap of the number of cis-acting elements in promoter regions, with color depth representing the number of elements. The total number and enrichment of elements in the *PgPR10* family were significantly higher than those in the *PgPR1* family, and MeJA, ABA and low-temperature response elements showed specific high distribution in *PgPR10*.

The promoter regions of members from both the *PgPR1* and *PgPR10* gene families contain diverse types of cis-acting regulatory elements, including those involved in light response, hormone response, abiotic stress response, and tissue development regulation. The types and distribution patterns of these elements exhibit both family-shared and family-specific characteristics, reflecting differences in expression regulation and functional specialization between the two families. As shown in Figure 5a, light response elements are the core shared elements, whereas hormone and stress response elements display family-specific distribution patterns. Light response elements are enriched in the promoters of all members of both families and represent the most abundant element type in each member, indicating that light signal regulation is a central pathway for the expression of both the *PgPR1* and *PgPR10* families. The expression of both families is significantly regulated by light signals, which aligns with the core mechanism by which plants rely on light signals to coordinate growth, development, and stress defense. Among hormone-related elements, methyl jasmonate (MeJA) response elements are widely distributed in both families and represent the predominant hormone-responsive element secondary to light response elements. Abscisic acid (ABA) response elements are more widely distributed and abundant in the *PgPR10* family, whereas salicylic acid (SA), gibberellin (GA), and auxin response elements are present in both families but show member-specific variation. The proportion of members containing both SA and low-temperature response elements is significantly higher in the *PgPR10* family than in the *PgPR1* family, reflecting divergence in their hormone regulatory networks. Among abiotic stress response elements, anaerobic response elements are ubiquitous in all members of both families. In contrast, low-temperature response elements are highly frequent in the *PgPR10* family, whereas only a few members of the *PgPR1* family contain this element, indicating that the *PgPR10* family possesses more extensive regulatory potential for cold stress responses. The member-specific distribution of these cis-acting regulatory elements serves as an important regulatory basis for differential expression and functional specialization within the two gene families, and their compositional differences reveal the molecular mechanisms underlying the functional divergence of the two families at the transcriptional regulation level.

The heatmap analysis of cis-regulatory elements (Figure 5b) further highlights the distinct distribution patterns between the two families: the total number and enrichment level of cis-acting elements in members of the *PgPR10* gene family are globally significantly higher than those in the *PgPR1* family.

### 3.5. Expression Pattern Analysis of PR1 and PR10 Genes in P. ginseng

**Figure 6:**
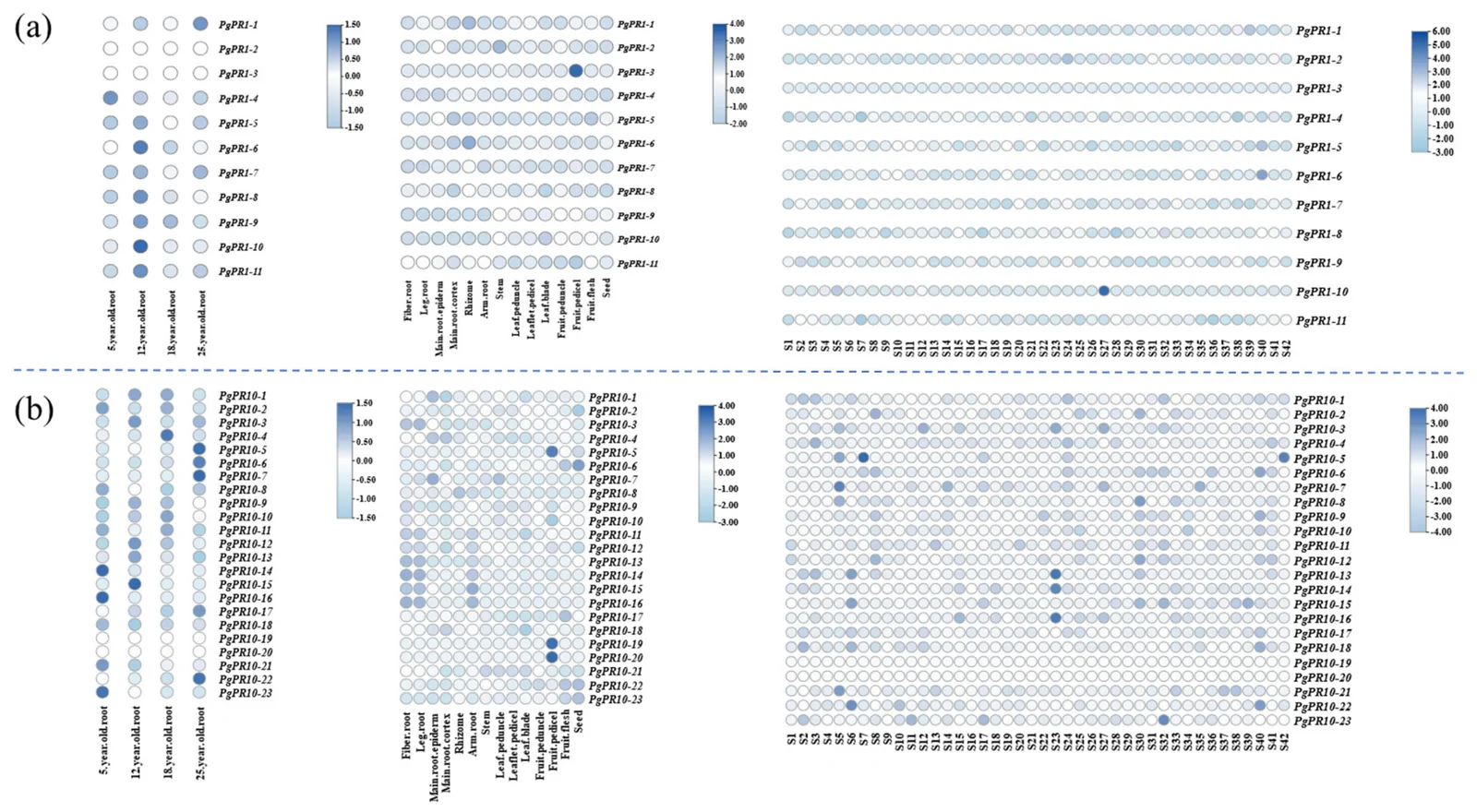
Heatmap analysis of spatiotemporal expression patterns of *PgPR1* and *PgPR10* genes in ginseng. (**a**) Heatmap of expression levels of *PgPR1* gene family in the 4 different ages (5, 12, 18, and 25 years old) of ginseng roots, 14 different tissues of 4-year-old ginseng, and 42 farmer’s cultivars of 4-year-old ginseng roots; (**b**) Heatmap of expression levels of *PgPR10* gene family in the 4 different ages (5, 12, 18, and 25 years old) of ginseng roots, 14 different tissues of 4-year-old ginseng, and 42 farmer’s cultivars of 4-year-old ginseng roots. The color depth of nodes represents the relative expression level of genes. The *PgPR1* family shows root-preferential expression, while the *PgPR10* family exhibits constitutive high expression in all tissues, and both families display significant expression differences among different ages and cultivars.

This study examined the expression levels of members of the *PgPR1* and *PgPR10* gene families in ginseng roots at different ages, different tissues and different farmers’ cultivars (Figure 6, Table S2), systematically analyzing the spatiotemporal expression patterns, inter-varietal expression variations, and tissue-specific expression profiles of the two *PR* families. As shown in the analysis results of Figure 6a,b, there was a marked divergence in the expression patterns between the two families, intra-family expression hierarchies, and functional specialization. The *PgPR1* family comprises 11 members, among which *PgPR1-8* is a highly expressed core member, showing high abundance across all tested conditions and peaking in 12-year-old roots. Most other *PgPR1* members are lowly expressed or expressed in a spatiotemporally specific or silenced manner, with only a few, such as *PgPR1-4* and *PgPR1-5*, displaying moderate to high expression that fluctuates with cultivation year. The overall expression level of the 23 members of the *PgPR10* family was significantly higher than that of the *PgPR1* family. *PgPR10-1*, *PgPR10-17*, and *PgPR10-18* are the dominant highly expressed members of this family, maintaining high expression in roots aged 5–25 years. The gradient of high, moderate, and low expression members within the family is more pronounced, and certain members, such as *PgPR10-5*, exhibit extremely strong cultivation year-specific high expression. Varietal-specific expression analysis indicated that *PgPR1-3* in the *PgPR1* family was completely silenced in all tested varieties, whereas *PgPR1-8* and *PgPR1-11* showed high expression in specific landraces. *PgPR10-19* and *PgPR10-20* are silenced members of the *PR10* family, and most lowly expressed members display varietal-specific expression features. The core members *PgPR10-1* and *PgPR10-18* maintained high expression levels across all 42 farmers’ cultivars, with inter-varietal expression differences spanning more than ten-fold, indicating stronger expression heterogeneity among cultivars.

The tissue-specific expression profiles of the two gene families further elucidate the core trends of functional divergence, with the expression patterns of both families closely associated with the growth, development, and organ specialization of *P. ginseng*. The *PgPR1* family exhibits a typical root-preferential expression pattern: *PgPR1-7* and *PgPR1-8* are highly expressed in fibrous roots and rootlets, whereas only *PgPR1-9* and *PgPR1-10* are specifically expressed in above-ground tissues, such as stems and leaves. The cortex of the taproot appears to be the primary site of expression for this family. In contrast, most members of the *PgPR10* family display high-level expression across all tissues examined. Core members, such as *PgPR10-1* and *PgPR10-18*, are highly expressed in various root tissues and maintain high expression levels in stems, petioles, and fruit flesh. Notably, *PgPR10-6* shows particularly high expression specifically in seeds, and *PgPR10-23* exhibits significantly higher expression in above-ground tissues than the above-ground-expressed members of the *PgPR1* family. These findings suggest that the *PgPR1* family is primarily involved in nutrient-growth processes in ginseng roots, whereas the expression profile of the *PgPR10* family, combined with its gene functional characteristics, indicates a potential role in the growth, development, and secondary metabolism of multiple organs throughout the ginseng plant.

### 3.6. Co-Expression Network Analysis of PR1 and PR10 Genes in P. ginseng

**Figure 7:**
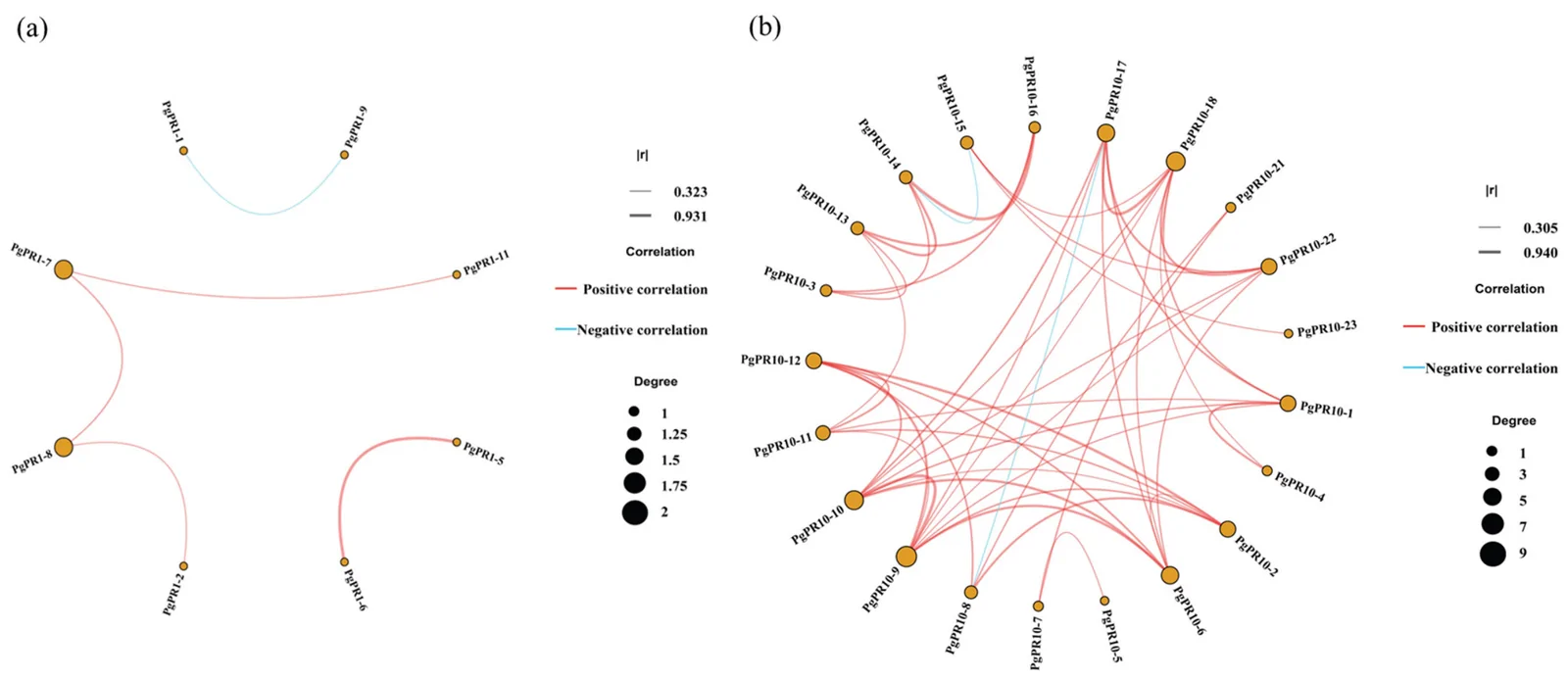
Co-expression network analysis of *PgPR1* and *PgPR10* gene families in ginseng. (**a**) Co-expression network of the *PgPR1* family constructed based on root expression data of 42 farmers’ cultivars of 4-year-old ginseng roots. Nodes represent genes, node size represents degree, edge thickness represents the absolute value of correlation coefficient (|*r*|) between genes, and red/blue represent positive/negative correlation, respectively; (**b**) co-expression network of the *PgPR10* family with a degree of 1–9 and complex structure.

Based on the gene expression data from 42 farmer cultivars of 4-year-old ginseng roots, co-expression networks were constructed for the *PgPR1* and *PgPR10* gene families. The strength of gene—gene associations was quantified using the absolute value of the correlation coefficient (|*r*|), with |*r*| values ranging from 0.323 to 0.931 for the *PgPR1* family and from 0.305 to 0.940 for the *PgPR10* family. Degree centrality was used to evaluate the core position of each gene in the network, thereby revealing the characteristics of coordinated or antagonistic expression regulation. The results are shown in Figure 7, indicating that the *PgPR1* family network has a simple structure, with degree values ranging from 1 to 2. Only two gene pairs exhibited negative correlations, whereas all other correlated gene pairs showed positive correlations. Overall, the coordinated expression among members of this family is weak, suggesting a relatively independent regulatory pattern. In contrast, the *PgPR10* family network is significantly more complex, with degree values ranging from 1 to 9. Most genes within this family are directly or indirectly connected, and the regulatory relationships are predominantly positive, forming a tightly interconnected internal regulatory network. The highly expressed core members of this family act as hubs in the network.

### 3.7. Screening of Candidate PR1 and PR10 Genes Associated with Ginsenoside Biosynthesis in Ginseng

**Figure 8:**
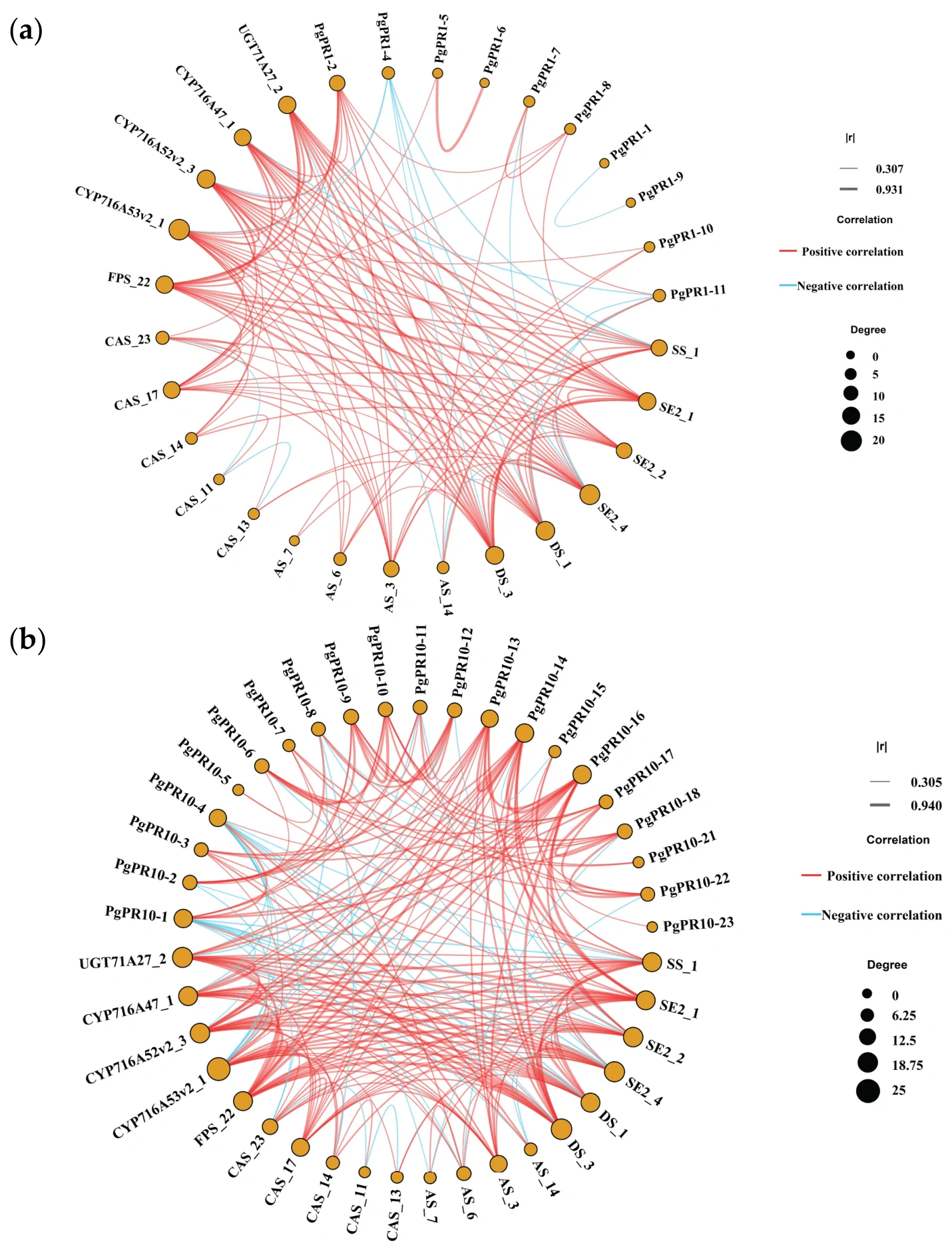
The co-expression network was constructed based on root expression data of 42 farmer cultivars of 4-year-old ginseng roots with a strict threshold of |r| ≥ 0.3 and *p* < 0.001. (**a**) Co-expression network analysis of the *PgPR1* gene family and key biosynthetic enzyme genes in ginseng; (**b**) Co-expression network analysis of the *PgPR10* gene family and key biosynthetic enzyme genes in ginseng. Nodes of different colors distinguish *PR* genes and key enzyme genes of ginsenoside biosynthesis. Node size represents degree, and edge thickness represents the absolute value of the correlation coefficient. PgPR1-5 and PgPR10-8 were the only *PR* family members significantly associated with all key enzyme genes of ginsenoside biosynthesis, forming a core regulatory module.

Based on the gene expression data from the 42 farmer cultivars of 4-year-old ginseng roots, this study screened candidate *PgPR1* and *PgPR10* genes associated with ginsenoside synthesis through co-expression network analysis (Table S3). The results showed (Figure 8) that the *PgPR1* family had a sparse network connection with weak co-expression among its members, whereas a tightly coordinated co-expression network was formed within the *PgPR10* family, which exhibited a stronger co-expression correlation with the key enzyme genes involved in ginsenoside biosynthesis. With a stringent threshold of *p* < 0.001, a core co-expression module integrating key genes from both ginsenoside biosynthesis and defense-related pathways was identified. Within this module, *PgPR1-5* and *PgPR10-8* are the only *PR* family members showing significant associations with all key enzyme genes involved in ginsenoside biosynthesis. These key enzyme genes span multiple critical metabolic steps, including precursor synthesis (*SS_1*, *SE2_1*, *SE2_4*, and *FPS_22*), oxidative modification (*CYP716A47_1*, *CYP716A52v2_3*, and *CYP716A53v2_1*), glycosylation (*UGT71A27_2*), and triterpenoid backbone formation (*DS_1*, *DS_3*, and *CAS_14*). Notably, *PgPR10-8* exhibited particularly strong coupling with the ginsenoside biosynthetic pathway, suggesting that it may act as a key hub gene mediating the crosstalk between plant defense responses and secondary metabolism regulation. This finding provides an important research target for further elucidating the molecular mechanisms by which PR proteins regulate ginsenoside biosynthesis.

### 3.8. Gene Expression Analysis of PgPR1-5, PgPR10-8 and Key Enzyme Genes Under MeJA Treatment

**Figure 9:**
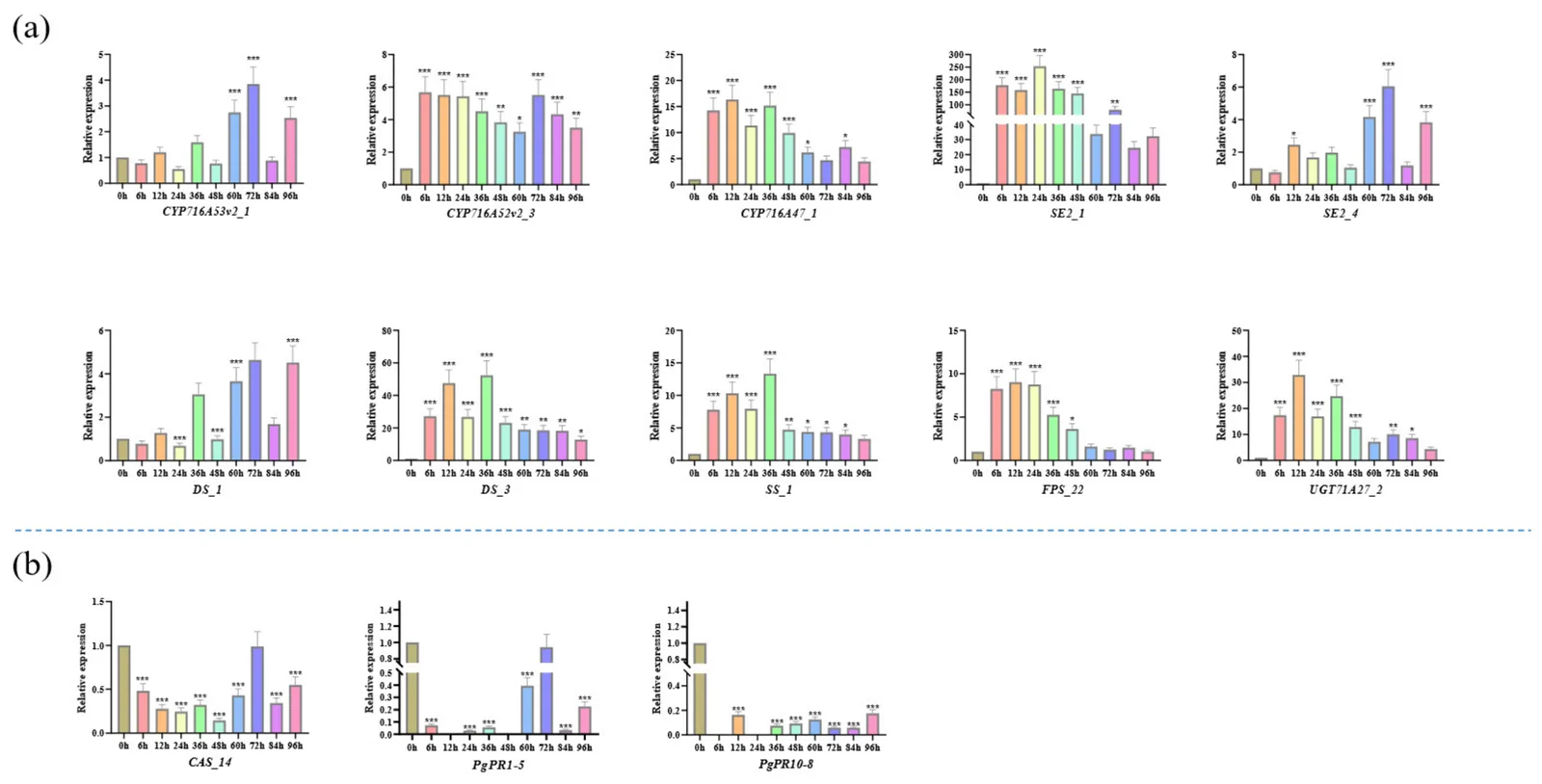
Dynamic changes in the relative expression levels of *PgPR1* and *PgPR10* gene families and key enzyme genes in ginseng adventitious roots under 200 μM MeJA treatment for 0–96 h (column chart). Data are presented as mean ± standard deviation (SD). The ordinate represents the relative expression level. (**a**) Temporal expression of key enzyme genes in the ginsenoside biosynthesis pathway under MeJA treatment. (**b**) Temporal expression of *PgPR1-5*, *PgPR10-8* and *CAS_14* under MeJA treatment, with their expression patterns being highly synchronous. * indicates a significant difference when *p* < 0.05, ** indicates a significant difference when *p* < 0.01, and *** indicates a significant difference when *p* < 0.001.

Gene expression analysis was performed on ginseng adventitious root samples subjected to MeJA treatment across a 0–96 h time course, with the 0 h time point serving as the initial control. This study focused on the expression dynamics of core genes from the *PgPR1* and *PgPR10* families *(PgPR1-5* and *PgPR10-8*) and key genes involved in ginsenoside biosynthesis. As illustrated in Figure 9, MeJA exerted a bidirectional temporal regulatory effect on the ginsenoside biosynthetic pathway. The temporal expression patterns of *PgPR1-5* and *PgPR10-8* were highly consistent with that of *CAS_14*, which encodes cycloartenol synthase responsible for phytosterol biosynthesis and competes with ginsenoside metabolism for the common precursor 2,3-oxidosqualene.

Key enzyme genes participating in precursor synthesis, oxidative modification, glycosylation modification, and triterpenoid backbone formation (including *DS_1* and *DS_3*) exhibited significantly higher relative expression under MeJA treatment compared with the 0 h control, with expression peaks appearing at different time points. In contrast, *CAS_14* was the only examined enzyme gene that was persistently significantly downregulated across the whole time series and maintained low, fluctuating transcript levels. Both *PgPR1-5* and *PgPR10-8* displayed significant expression changes relative to the control, although their relative expression values did not rise substantially above 1. *PgPR10-8* maintained a consistently low expression level, whereas *PgPR1-5* formed a slight expression peak at 72 h followed by a rapid decrease.

Overall, the expression profiles of the two *PgPR1-5* and *PgPR10-8* genes were highly synchronized with *CAS_14*. We hypothesize that these *PR* genes may participate in MeJA-mediated metabolic flux redistribution at the triterpenoid synthesis branch point dominated by *CAS_14*. This observation provides valuable clues for unraveling the molecular mechanism through which PR proteins modulate ginsenoside biosynthesis under MeJA signaling.

## 4. Discussion

This study, focusing on *P. ginseng*, identified 34 members of the *PR1*/*PR10* gene families (11 *PgPR1s* and 23 *PgPR10s*) in the ginseng genome. Compared with the model plant *A. thaliana*, the *PgPR1* family in ginseng exhibits a relatively compact size (in *A. thaliana*, *PR1* and *PR1-like* genes form a multigene family), whereas the *PgPR10* family in ginseng has undergone significant expansion (the PR10 family is considerably smaller in *A. thaliana*). This difference reflects the selective expansion of PR10 proteins with their multifunctional roles in ligand binding, stress response, and metabolic regulation in *P. ginseng*, a perennial allotetraploid medicinal plant, as a result of long-term adaptation to the understory-shaded environment. Notably, 152 *PR* genes (including multiple PR10 members) have been identified in *P. notoginseng*, indicating that *PR* gene family expansion is a common phenomenon in Araliaceae medicinal plants [30]. However, the extent of expansion appears to be closely associated with the complexity of secondary metabolism and the ecological adaptation strategies of each species. This finding provides a novel perspective for understanding the coordinated regulation of defense and secondary metabolism in perennial medicinal plants.

This evolutionary characteristic of the *PR* gene family and its functional differentiation are closely associated with species’ survival strategies [31]. In the model plant *A. thaliana*, family members such as PR-6 (proteinase inhibitors), PR-12 (plant defensins), PR-13 (thionins), and PR-14 (lipid transfer proteins) mainly exist as small-molecule peptides and participate in biotic stress defense through direct antimicrobial activity or signal transduction [32]. By contrast, *GmPR10-1* in soybean improves plant tolerance to abiotic stresses such as drought by modulating the ABA signaling pathway, thus regulating stomatal movement and antioxidant enzyme activity [33]. In *P. notoginseng*, the *PR* gene family has undergone remarkable expansion with 152 members, among which *PnPR4* acts as a core component conferring root rot resistance mainly by directly inhibiting pathogen growth [34]. In tomato, the *SlPR-1* gene is significantly upregulated under drought stress and participates in abiotic stress responses [35]. *PdePR1_10* from poplar has been characterized as a key defense marker gene against leaf rust [36]. Moreover, overexpression of the tobacco *Osmotin* (PR-5) gene in carrot markedly enhances drought resistance [37]. In contrast, the *PR* gene complement in *P. ginseng* remains relatively compact, comprising only 34 *PR1/PR10* members. Notably, its evolutionary differentiation is specialized towards the coordination of defense and primary/secondary metabolism. The *PgPR1* subfamily maintains high sequence conservation and exerts basal antibacterial functions via root-specific expression. Conversely, the *PgPR10* subfamily has undergone distinct species-specific expansion. Equipped with a Bet-v-1-like ligand-binding domain and an array of hormone-responsive cis-elements, *PgPR10* members serve as key integrators linking stress responses to metabolic regulation. This strategy reflects the unique adaptive mechanism of ginseng, where constitutive chemical defense (mediated by high ginsenoside levels) is coupled with inducible regulatory networks. This stands in contrast to *PR* genes from other plant species, which typically exhibit specialized functions confined to single stress response pathways. *PR* genes are conventionally recognized as vital participants responding to biotic and abiotic stresses. This study moves beyond this paradigm. Through stringent co-expression network analysis (|*r*| ≥ 0.3, *p* < 0.001), we identified, for the first time, *PgPR1-5* and *PgPR10-8* as core *PR* gene family members that are specifically and strongly associated with key ginsenoside biosynthetic enzyme genes. This highly specific association points to a novel direction for expanding the functional repertoire of PR proteins, distinguishing it from the direct stress-combating mechanisms observed for PR proteins in other plant species.

MeJA serves as a core signaling molecule in plant defense responses, typically activating the expression of pathogenesis-related (PR) proteins to directly participate in pathogen suppression or to induce systemic resistance [38,39]. MeJA acts as a core signaling molecule in plant defense responses, typically upregulating the expression of the *PR* genes to directly inhibit pathogens or induce systemic resistance [40,41]. However, the present study revealed that exogenous MeJA exerts a complex, bidirectional regulatory effect on the ginsenoside biosynthetic pathway in *P. ginseng*. On the one hand, it significantly upregulates several key enzyme genes involved in precursor synthesis and modification (e.g., *FPS_22*, *UGT71A27_2*). On the other hand, *CAS_14*, which encodes cycloartenol synthase functioning at the phytosterol synthetic branch, was markedly suppressed throughout the treatment period. Notably, the expression profiles of multiple biosynthetic genes exhibited non-monotonic fluctuations over the treatment time series, such as the variation in *DS_1* at 36 h, oscillations of *CAS_14* at 72 h, and the obvious decline of *SE2_4* and *DS_1* at 84 h. Such dynamic fluctuations are frequently observed in secondary metabolic pathways under elicitor treatment. This phenomenon can be mainly attributed to endogenous metabolic feedback regulation: continuous accumulation of pathway intermediates may trigger transient transcriptional inhibition of biosynthetic genes. Meanwhile, progressive signal reprogramming and complex hormone crosstalk after MeJA application also contribute to oscillating expression patterns rather than simple continuous upregulation. Natural biological variation among independently cultured adventitious root samples may also partly account for minor fluctuations. As a key branch-point enzyme, *CAS_14* competes with ginsenoside synthetic enzymes for the common upstream substrate 2,3-oxidosqualene, thereby mediating metabolic flux distribution between triterpene and phytosterol pathways. Notably, two *PR* family genes, *PgPR1-5* and *PgPR10-8*, exhibited expression trends highly consistent with *CAS_14*. Both genes maintained relatively low expression levels and were not induced by MeJA throughout the time-course treatment. This tightly synchronized expression pattern implies that *CAS_14* and these specific *PR* genes may be co-repressed by shared upstream regulatory modules under MeJA signaling. We speculate that this bidirectional metabolic regulation represents a sophisticated adaptive strategy in perennial ginseng. MeJA simultaneously promotes the expression of ginsenoside-modifying enzymes and restricts the competing phytosterol branch, which may redirect metabolic flux toward ginsenoside production while avoiding excessive accumulation of upstream intermediates and unnecessary metabolic consumption. This delicate transcriptional balance reveals the flexible and precise regulation of specialized metabolism in ginseng in response to defense signals. Further investigation is still required to clarify whether PR genes function merely as co-expressed markers or actively participate in the feedback modulation of triterpene metabolic flux during MeJA-induced defense responses.

Distribution patterns of cis-acting elements determine the transcriptional regulatory characteristics of *PR* genes. The promoter of soybean *GmPR10-1* harbors a GCC-box element that can be directly bound and activated by *GmERF113*, thereby participating in the ABA signaling pathway [42]. Promoters of the *PR* genes in *P. notoginseng* are enriched with defense- and stress-responsive cis-elements, supporting their functional roles in disease resistance [43]. In *Arabidopsis*, JA/ET-responsive elements in *PR* gene promoters are crucial for their involvement in defense signal transduction [44]. Promoters of *PgPR* genes also exhibit family-specific characteristics. Both the *PgPR1* and *PgPR10* families are enriched with light- and MeJA-responsive elements, reflecting conserved transcriptional features within *PR* genes in ginseng. In contrast, the *PgPR10* family is additionally enriched with ABA- and low-temperature-responsive elements, and the total number of cis-elements is significantly higher than that in the *PgPR1* family, facilitating flexible transcriptional responses to diverse environmental stimuli. In terms of gene structure, the CAP_PR-1 domain of the *PgPR1* family and the Bet-v-1 domain of the *PgPR10* family are singular in type but functionally specialized. The Bet-v-1 domain may participate in phytohormone signal transduction or small-molecule transport by binding to metabolites, such as fatty acids and flavonoids, providing a structural basis for the multifunctionality of PR10 proteins in ginseng, a feature that has not been widely documented in *PR* gene studies in other plants.

This study, through bioinformatic analyses and expression validation, preliminarily explores the potential links between *PgPR1-5* and *PgPR10-8* and ginsenoside biosynthesis. However, several limitations remain. First, the co-expression network only reflects expression associations; direct molecular interactions between *PgPR1-5* and *PgPR10-8* with the key enzyme genes have not been experimentally verified (e.g., via ChIP-qPCR or yeast one-hybrid assays). Second, the upstream signaling pathways connecting *PR* genes to ginsenoside biosynthesis remain unclear. Third, the ligand-binding specificity of the Bet-v-1 domain in *PgPR10* proteins is lacking in-depth analysis. Future research could focus on three areas: (1) employing gene interference or overexpression techniques to define the impact of *PgPR1-5* and *PgPR10-8* on ginsenoside content and defense responses; (2) elucidating the interactions between *PR* genes and transcription factors to clarify their regulatory hierarchy within the MeJA signaling pathway; and (3) combining protein-interaction and metabolite-binding assays to uncover how PgPR10 proteins may participate in triterpenoid metabolism. Furthermore, the *PgPR1-5* and *PgPR10-8* genes identified in this study provide promising candidates for subsequent functional research on *Panax ginseng* and serve as a reference for functional studies of PR genes in other medicinal plants, such as *P. notoginseng*. In summary, this work systematically characterizes the evolutionary and differentiation features of the *PgPR1* and *PgPR10* gene families in ginseng, identifies coordinated expression patterns of core PR members alongside ginsenoside biosynthetic genes and their bidirectional response pattern under MeJA signaling, enriches the functional diversity of the *PR* gene family, and provides a theoretical foundation for understanding the coordination between defense and secondary metabolism in perennial medicinal plants.

## 5. Conclusions

This study focused on *P. ginseng* and systematically investigated the molecular characteristics, evolutionary patterns, and expression correlations with key ginsenoside biosynthetic enzyme genes of the *PgPR1* and *PgPR10* gene families through multiple experimental approaches, including transcriptomic and genomic identification, evolutionary analysis, construction of a co-expression network, and MeJA-induced expression validation. A genome and transcriptome systematic identification of the *PgPR1* and *PgPR10* gene families in ginseng was conducted, yielding a total of 34 family members (11 *PgPR1*s and 23 *PgPR10*s).

We screened core PR protein family members PgPR1-5 and PgPR10-8, which are closely associated with key ginsenoside biosynthetic enzyme genes. Stringent co-expression network analysis (|*r*| ≥ 0.3, *p* < 0.001) confirmed that they are the only PR protein family members significantly correlated with key ginsenoside biosynthetic enzyme genes spanning precursor synthesis, oxidative modification, glycosylation modification, and triterpenoid backbone synthesis. This establishes a specific expression linkage between PR proteins and the ginsenoside biosynthesis pathway, challenging the traditional view that PR proteins are involved only in stress responses. The coordinated expression patterns of *PgPR1-5* and *PgPR10-8* with key ginsenoside biosynthetic enzyme genes under MeJA induction were elucidated. MeJA exerts bidirectional temporal regulation on the ginsenoside pathway: key enzyme genes involved in precursor synthesis and oxidative modification (e.g., *FPS_22*, *UGT71A27_2*, *CYP716A53v2_1*) were significantly upregulated, whereas the key triterpenoid backbone synthase gene *CAS_14* was specifically downregulated. The temporal expression profiles of *PgPR1-5* and *PgPR10-8* were highly synchronized with those of *CAS_14*, maintaining low expression levels throughout with no significant upregulation. This confirms their coordinated response with *CAS_14* under MeJA regulation and provides direct expression-level evidence for further investigation of their regulatory roles.

In summary, for the first time, this study systematically elucidated the molecular characteristics and evolutionary patterns of the *PgPR1* and *PgPR10* gene families, identified core *PR* genes associated with ginsenoside biosynthesis, and revealed the expression correlation between these core *PR* genes and key enzyme genes, as well as their coordinated response under MeJA regulation. This work expands the understanding of the functional diversity of the *PR* gene family, provides important bioinformatic insights and candidate targets for further investigation of the molecular mechanisms by which PR proteins regulate ginsenoside biosynthesis, and offers theoretical foundations and genetic resources for molecular breeding aimed at developing *P. ginseng* varieties with high ginsenoside content and enhanced stress resistance.

## Figures and Tables

**Table 1:** Details of gene primers used in this study for RT-qPCR.

Number	Primer Name	Primer Sequence	Product Size
1	Action 1-F	TGGCATCACTTTCTACAACG	150 bp
	Action 1-R	TTTGTGTCATCTTCTCCCTGTT	
2	PgPR1-5-F	GGTTGCATCGGAGAGTCCTT	115 bp
	PgPR1-5-R	CCCACCATCTGGCGTACTTT	
3	PgPR10-8-F	GCCGCCATCTCCTTCAATCA	159 bp
	PgPR10-8-R	GCATCAAGAGGCATGCCAAA	
4	CAS_14-F	GATGGACAAGGGGCAATGGA	143 bp
	CAS_14R	ATCTCTGGGGGCAGAGGATT	
5	CYP716A53v2_1-F	CGCTGACTTTTGAGTTGGCC	155 bp
	CYP716A53v2_1-R	GGCCTTGATCCCACGGTTAA	
6	CYP716A52v2_3-F	GAGGGAGTCTACCAGGAGCA	121 bp
	CYP716A52v2_3-R	GTCTCAGCACTTCACAGGCT	
7	CYP716A47_1-F	ACATAGAGGCCGGACTCCTT	105 bp
	CYP716A47_1-R	TCACCGCCTCAACCTCTCTA	
8	SE2_1-F	GTCCGACAGAGCAACAGTCA	113 bp
	SE2_1-R	ACAGAAGCATGCCAGCTGAT	
9	SE2_4-F	TAATCAAAGCATGCGTCGCG	80 bp
	SE2_4-R	TTGGCTGGTGCGCTATACAA	
10	DS_1-F	AGTATCCACTTCCGGCCTCT	151 bp
	DS_1-R	ATGAGGATGGTGGATGGGGA	
11	DS_3-F	AGTATCCACTTCCGGCCTCT	150 bp
	DS_3-R	TGAGGATGGTGGATGGGGAT	
12	SS_1-F	TGGGGGCAATTCTGAAGCAT	160 bp
	SS_1-R	TGTTGAATGACGAGGCCGAA	
13	FPS_22-F	CTGGATTGCTTTGGTGCACC	166 bp
	FPS_22-R	CTTTTGCTACAGAGGCCGGA	
14	UGT71A27_2-F	AGGTGCAGAGAGTCAAAGGC	169 bp
	UGT71A27_2-R	CAAGGATCACAGCAGCCTCA	

## Data Availability

All ginseng samples can be accessed upon request from the corresponding author.

## Supplementary Materials

The following supporting information can be downloaded at https://www.mdpi.com/article/10.3390/life16091446/s1: Table S1: Basic information of the *PR1* and *PR10* gene family in *Panax ginseng*. Table S2: The TPM expressions of the *PgPR1* and *PgPR10* genes in the 4 different ages (5, 12, 18, and 25 years old) of ginseng roots, 14 different tissues of 4-year-old ginseng, and 42 farmer cultivars of 4-year-old ginseng roots. Table S3: GO functional annotations of key enzyme genes involved in the ginsenoside biosynthetic pathway in *Panax ginseng*.
